# Low-Molecular-Weight Fish Collagen Peptide Enhances Hair Regrowth via Activation of Proliferative Signaling and Suppression of Inhibitory Pathways

**DOI:** 10.3390/md24070233

**Published:** 2026-07-03

**Authors:** Hyelim Kim, Yeonhwa Lee, Seong-Hoo Park, Hyunyoung Choi, Joon Sung Yang, Kyung Seok Kim, Woojin Jun

**Affiliations:** 1 Department of Medical Nutrition, AgeTech-Service Convergence Major, Graduate School of East-West Medical Science, Kyung Hee University, Yongin 17104, Republic of Korea; hlimkim46@khu.ac.kr; 2 Research Institute of Medical Nutrition, Kyung Hee University, Seoul 02447, Republic of Korea; phoo3166@khu.ac.kr (S.-H.P.); hyun00807@khu.ac.kr (H.C.); 3 Division of Food and Nutrition, Chonnam National University, Gwangju 61186, Republic of Korea; qazwsx917@naver.com; 4 Research&Department, Geltech, Busan 46752, Republic of Korea; joonyang@geltech.co.kr; 5 New Ingredient Development Research Center, Suheung, Gwacheon 13840, Republic of Korea; kskim1@suheung.com; 6 Research Institute for Human Ecology, Chonnam National University, Gwangju 61186, Republic of Korea

**Keywords:** collagen peptides, hair growth, Wnt/β-catenin

## Abstract

Collagen peptides have been widely studied for their beneficial effects on skin health; however, their potential role in hair growth remains insufficiently explored. This study aimed to investigate the effects of orally administered low-molecular-weight fish collagen peptide (SH-GT) on hair regrowth and its underlying mechanisms in a hair-removed C57BL/6J mouse model. Mice were administered SH-GT (100, 300, or 600 mg/kg body weight) or a positive control (Pansidil, 400 mg/kg) daily for 28 days. SH-GT significantly enhanced hair regrowth, as evidenced by the increased hair growth area. Histological analysis revealed increased dermal thickness and visible hair follicle structures in SH-GT-treated groups. At the molecular level, SH-GT upregulated proliferation-related proteins, including PCNA and Cyclin D1, and activated Wnt/β-catenin signaling. In addition, SH-GT enhanced PI3K/Akt/mTOR signaling, suggesting improved cellular growth and survival. Conversely, SH-GT suppressed hair growth inhibitory pathways by reducing BMP4 expression and decreasing Smad phosphorylation. Furthermore, SH-GT increased the mRNA expression of growth factors such as IGF-1, HGF, VEGF, EGF, and FGF7. In conclusion, SH-GT promotes hair regrowth by simultaneously activating proliferation-related signaling pathways and suppressing inhibitory mechanisms, thereby improving the dorsal skin microenvironment associated with hair regrowth. These findings suggest that SH-GT may serve as a promising functional ingredient for improving hair growth.

## 1. Introduction

Hair plays an important role in thermoregulation, protection, and appearance, and its loss or thinning can significantly affect quality of life. Hair growth is a dynamic and cyclic process consisting of three distinct phases: anagen (growth), catagen (regression), and telogen (resting). Among these, the anagen phase is critical for active hair follicle proliferation and elongation of the hair shaft. Dysregulation of this cycle, particularly premature transition to the catagen or telogen phase, is a major cause of hair loss. Therefore, strategies that promote anagen entry and prolong its duration are considered key approaches for improving hair growth [1,2,3,4].

Hair follicle cycling is tightly regulated by complex signaling networks that coordinate cellular proliferation, differentiation, and apoptosis within the follicular microenvironment. Among these, the Wnt/β-catenin signaling pathway plays a central role in initiating the anagen phase and promoting hair follicle development by activating keratinocyte proliferation and stem cell differentiation. Activation of β-catenin signaling induces downstream targets such as Cyclin D1, thereby facilitating cell cycle progression [5,6,7]. In addition, the PI3K/Akt/mTOR pathway is critically involved in regulating cell survival, growth, and protein synthesis in hair follicle cells, further supporting hair regeneration [8,9]. Conversely, hair growth is negatively regulated by inhibitory pathways such as transforming growth factor-β (TGF-β) and bone morphogenetic protein (BMP) signaling. Activation of Smad proteins downstream of these pathways induces catagen transition and suppresses hair follicle proliferation [10]. Therefore, modulation of both stimulatory and inhibitory signaling pathways is essential for maintaining proper hair follicle cycling and promoting hair growth.

Collagen, a major structural protein in the extracellular matrix, has gained increasing attention as a functional ingredient due to its diverse biological activities. Oral intake of collagen peptides has been widely reported to improve skin health by enhancing dermal thickness, elasticity, and hydration [11,12]. In addition, collagen supplementation has been associated with beneficial effects on bone metabolism, joint health, and muscle function. These effects are thought to be mediated by the absorption of bioactive peptides, which can influence cellular signaling pathways and extracellular matrix remodeling [13,14]. Compared with conventional high-molecular-weight collagen, low-molecular-weight collagen peptides have several advantages, including improved solubility, digestibility, and intestinal absorption. In particular, enzymatic hydrolysis converts collagen into smaller oligopeptides that can be more readily absorbed, and collagen-derived hydroxyproline-containing di- and tripeptides have been detected in human plasma after oral intake [15,16]. These absorbed peptides may exert biological effects by modulating cellular signaling pathways and extracellular matrix remodeling. Therefore, low-molecular-weight collagen peptide technology is considered a useful approach for enhancing the functional applicability of collagen-derived bioactive peptides [13,15].

Collagen can be obtained from various sources, including bovine, porcine, chicken, and marine organisms. Among these, fish-derived collagen has attracted attention as a functional food material because it has fewer religious and cultural restrictions and a lower risk of mammalian disease-related concerns than land animal-derived collagen. In addition, fish scales are collagen-rich by-products generated during fish processing and can be used as a practical source for producing collagen peptides [17,18]. Based on these advantages, tilapia (Oreochromis genus) fish scale collagen was selected as the source material for SH-GT because it represents a safe, practical, and marine-derived collagen source suitable for enzymatic production of low-molecular-weight collagen peptides. Tilapia fish scale collagen can be converted into low-molecular-weight oligopeptides through enzymatic hydrolysis, and our previous study identified the octapeptide Val-Gly-Pro-Hyp-Gly-Pro-Ala-Gly as a characteristic low-molecular-weight collagen peptide derived from this source [13]. Since hair follicles are embedded in the dermis and their growth is closely associated with the extracellular matrix, dermal thickness, and dermal papilla cell activity, tilapia-derived low-molecular-weight fish collagen peptide may provide a suitable bioactive peptide source for supporting hair follicle function and hair regrowth [10,13].

Despite extensive studies on the functional properties of collagen, research focusing on its role in hair growth remains limited. Given that hair follicles are embedded within the dermal matrix and rely on extracellular structural support as well as growth factor-mediated signaling [19], collagen peptides may contribute to hair follicle function by modulating the dermal microenvironment and associated signaling pathways. Furthermore, the potential of orally administered low-molecular-weight collagen peptides to regulate hair follicle-related signaling pathways, including the Wnt/β-catenin, PI3K/Akt/mTOR, and BMP/Smad pathways, has not been fully elucidated. In particular, this study focused on the potential of orally administered collagen peptides to improve the hair follicle microenvironment rather than targeting androgen-related therapeutic mechanisms. Therefore, in the present study, we investigated the effects of orally administered low-molecular-weight fish collagen peptide (SH-GT) on hair regrowth in a hair-removed mouse model.

## 2. Results

### 2.1. SH-GT Promotes Hair Regrowth in a Hair-Removed Mouse Model

**Figure 1:**
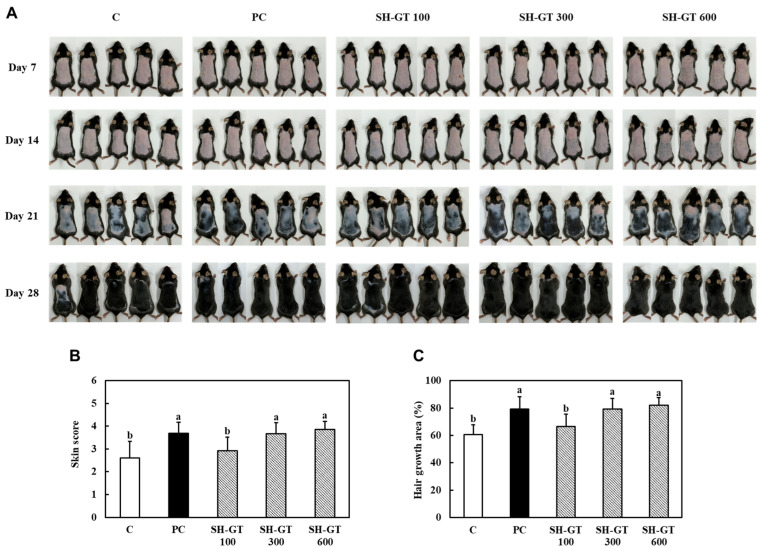
Effect of SH-GT on hair regrowth in hair-removed C57BL/6J mice. (**A**) Representative dorsal skin images showing hair regrowth on experimental days 7, 14, 21, and 28, corresponding to 0, 7, 14, and 21 days after hair removal, respectively, and (**B**) skin score and (**C**) hair growth area (%) were evaluated on experimental day 28. C (Control; hair removal + vehicle), PC (Positive control; hair removal + Pansidil 400 mg/kg b.w.), SH-GT 100 (hair removal + SH-GT 100 mg/kg b.w.), SH-GT 300 (hair removal + SH-GT 300 mg/kg b.w.), and SH-GT 600 (hair removal + SH-GT 600 mg/kg b.w.). Test materials were orally administered once daily for 28 days. All values are presented as mean ± SD (*n* = 9). Different letters indicate significant differences among groups as determined by one-way ANOVA followed by Duncan’s multiple range test (*p* < 0.05).

To evaluate the effect of SH-GT on hair regeneration, visual changes in dorsal skin were monitored over 28 days following hair removal (Figure 1A). While limited hair regrowth was observed in the control group, mice treated with SH-GT exhibited visibly accelerated hair regeneration, particularly at higher doses. By Day 21, the SH-GT 300 and 600 groups showed markedly darker skin and more extensive hair coverage compared to the control group, indicating earlier entry into the anagen phase. At Day 28, the dorsal skin of SH-GT-treated mice was almost completely covered with newly grown hair comparable to or exceeding that of the positive control group.

Consistent with these observations, quantitative analysis revealed that the skin color score was significantly increased in the SH-GT 300 and 600 groups compared to the control group (*p* < 0.05; Figure 1B). Similarly, the hair growth area (%) was significantly elevated in SH-GT-treated groups, with SH-GT 300 and SH-GT 600 showing comparably high increases that reached levels similar to the positive control group (*p* < 0.05; Figure 1C). These results demonstrate that oral administration of SH-GT effectively promotes hair regrowth in vivo, suggesting its potential as a functional ingredient for improving hair growth.

Body weight, cage-level food consumption, FER, and major organ weights were evaluated to assess the general health status of mice during the experimental period. Although food consumption was slightly increased in the SH-GT 300 and SH-GT 600 groups compared with the control group, no significant differences were observed in initial body weight, final body weight, body weight gain, FER, or liver, kidney, and spleen weights among the experimental groups. In addition, no abnormal clinical signs were detected by visual observation during the experimental period (Table S1).

### 2.2. SH-GT Enhances Dermal Thickness and Visible Hair Follicle Structures in a Hair-Removed Mouse Model

**Figure 2:**
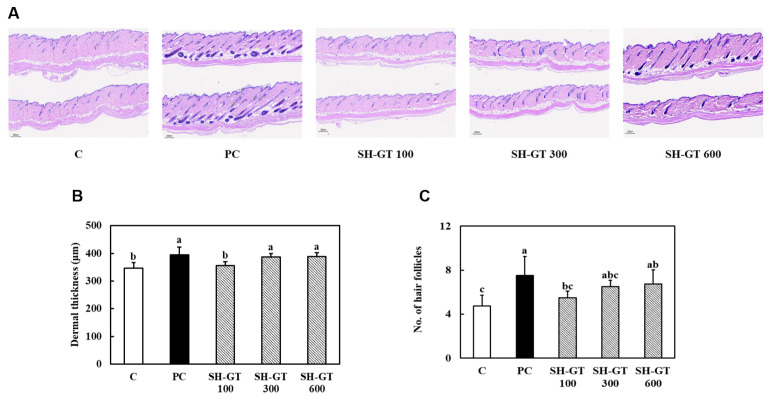
Effect of SH-GT on histological changes in dorsal skin of hair-removed C57BL/6J mice. (**A**) Representative hematoxylin and eosin (H&E)-stained longitudinal sections of dorsal skin (scale 200 μm), (**B**) dermal thickness (µm), and (**C**) number of hair follicles. C (Control; hair removal + vehicle), PC (Positive control; hair removal + Pansidil 400 mg/kg b.w.), SH-GT 100 (hair removal + SH-GT 100 mg/kg b.w.), SH-GT 300 (hair removal + SH-GT 300 mg/kg b.w.), and SH-GT 600 (hair removal + SH-GT 600 mg/kg b.w.). Test materials were orally administered once daily for 28 days. All values are presented as mean ± SD (*n* = 9). Different letters indicate significant differences among groups as determined by one-way ANOVA followed by Duncan’s multiple range test (*p* < 0.05).

To further confirm the hair growth-promoting effect of SH-GT at the histological level, dorsal skin tissues were analyzed by H&E staining (Figure 2A). The control group exhibited relatively thin dermal layers with fewer and less developed hair follicles. In contrast, SH-GT-treated groups showed visibly thickened dermal structures and increased density of hair follicles, particularly at higher doses.

Quantitative analysis demonstrated that dermal thickness was significantly increased in the SH-GT 300 and 600 groups compared to the control group, and these groups reached levels that were not significantly different from the positive control group (*p* < 0.05; Figure 2B). For hair follicle number, Sh-GT treatment showed a gradual increasing tendency rather than a statistically significant different from the control group, whereas the SH-GT 600 group showed a significant increase compared with the control group and reached a level that was not significantly different from that of the positive control group (*p* < 0.05; Figure 2C). These results suggest that high-dose SH-GT produced histological improvement comparable to Pansidil, particularly in dermal thickness and hair follicle number. These findings indicate that SH-GT promotes structural remodeling of dorsal skin by enhancing dermal thickness and increasing visible hair follicle structures, thereby supporting its role in facilitating hair regrowth.

### 2.3. SH-GT Activates Proliferation-Related Signaling Pathways in Dorsal Skin Tissues of a Hair-Removed Mouse Model

**Figure 3:**
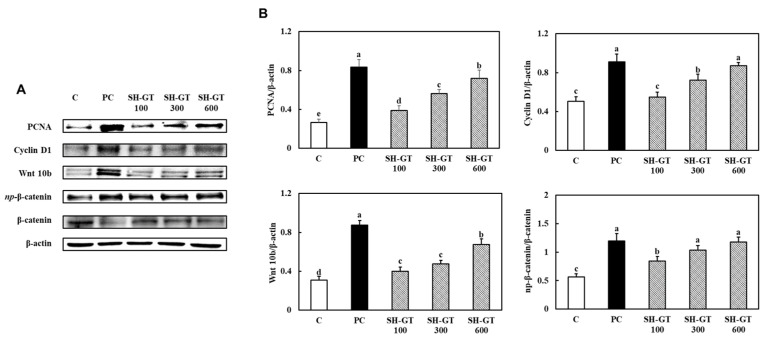
Effect of SH-GT on proliferation-related protein expression in dorsal skin of hair-removed C57BL/6J mice. (**A**) Representative Western blot images showing protein expression of PCNA, Cyclin D1, Wnt10b, non-phosphorylated (active) β-catenin (np-β-catenin), and total β-catenin and (**B**) quantitative analysis of these proteins. C (Control; hair removal + vehicle), PC (Positive control; hair removal + Pansidil 400 mg/kg b.w.), SH-GT 100 (hair removal + SH-GT 100 mg/kg b.w.), SH-GT 300 (hair removal + SH-GT 300 mg/kg b.w.), and SH-GT 600 (hair removal + SH-GT 600 mg/kg b.w.). Test materials were orally administered once daily for 28 days. All values are presented as mean ± SD (*n* = 9). Different letters indicate significant differences among groups as determined by one-way ANOVA followed by Duncan’s multiple range test (*p* < 0.05).

To investigate whether SH-GT promotes hair regrowth through modulation of proliferation-related signaling, the expression of key proteins associated with cell proliferation and Wnt/β-catenin signaling was evaluated in dorsal skin tissues (Figure 3A). The expression of proliferating cell nuclear antigen (PCNA), a marker of cell proliferation, was significantly increased in SH-GT-treated groups in a dose-dependent manner, with the highest level observed in the SH-GT 600 group. Similarly, Cyclin D1 expression was markedly upregulated following SH-GT administration, indicating enhanced cell cycle progression. In addition, SH-GT treatment significantly increased the protein expression of Wnt10b compared to the control group. Consistent with this, the level of active β-catenin (np-β-catenin/β-catenin ratio) was also significantly elevated in SH-GT-treated groups compared to the control group (*p* < 0.05; Figure 3B).

These results suggest that SH-GT enhances proliferation-related signaling in dorsal skin tissues by activating the Wnt/β-catenin pathway and promoting cell cycle-related protein expression, thereby contributing to accelerated hair regrowth.

### 2.4. SH-GT Enhances PI3K/Akt/mTOR Signaling in a Hair-Removed Mouse Model

**Figure 4:**
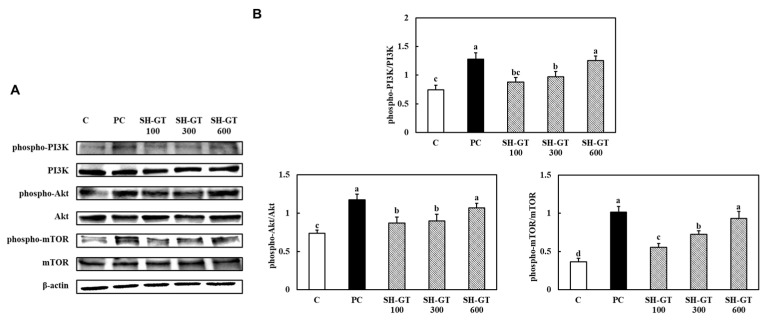
Effect of SH-GT on PI3K/Akt/mTOR signaling in dorsal skin of hair-removed C57BL/6J mice. (**A**) Representative Western blot images showing protein expression of phospho-PI3K, PI3K, phospho-Akt, Akt, phospho-mTOR, and mTOR and (**B**) quantitative analysis of these proteins. C (Control; hair removal + vehicle), PC (Positive control; hair removal + Pansidil 400 mg/kg b.w.), SH-GT 100 (hair removal + SH-GT 100 mg/kg b.w.), SH-GT 300 (hair removal + SH-GT 300 mg/kg b.w.), and SH-GT 600 (hair removal + SH-GT 600 mg/kg b.w.). Test materials were orally administered once daily for 28 days. All values are presented as mean ± SD (*n* = 9). Different letters indicate significant differences among groups as determined by one-way ANOVA followed by Duncan’s multiple range test (*p* < 0.05).

We confirmed the activation of the PI3K/Akt/mTOR signaling pathway in dorsal skin tissues to elucidate the molecular mechanisms underlying SH-GT-induced hair regrowth (Figure 4A). The phosphorylation level of PI3K (phospho-PI3K/PI3K) was significantly increased in SH-GT-treated groups, with the SH-GT 600 group showing a level comparable to the positive control. SH-GT treatment significantly elevated the phosphorylation of Akt, indicating enhanced downstream signaling associated with cell survival and proliferation. Moreover, the phosphorylation of mTOR, a key regulator of cellular growth and protein synthesis, was also significantly upregulated in SH-GT-treated groups in a dose-dependent manner. Notably, the SH-GT 600 group exhibited the highest activation among all treatment groups (*p* < 0.05; Figure 4B).

These findings indicate that SH-GT promotes hair growth not only by activating Wnt/β-catenin signaling but also by enhancing PI3K/Akt/mTOR-mediated pathways, thereby supporting proliferation- and growth-related signaling in the dorsal skin microenvironment.

### 2.5. SH-GT Suppresses Hair Growth Inhibitory Signaling Pathways in a Hair-Removed Mouse Model

**Figure 5:**
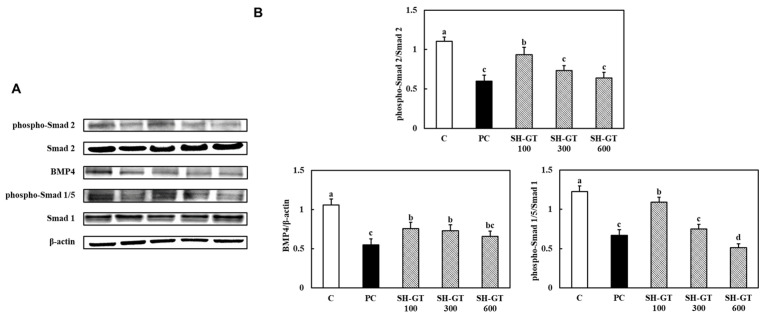
Effect of SH-GT on hair growth inhibitory signaling in dorsal skin of hair-removed C57BL/6J mice. (**A**) Representative Western blot images showing protein expression of phospho-Smad2, Smad2, BMP4, phospho-Smad1/5, and Smad1 and (**B**) quantitative analysis of these proteins. C (Control; hair removal + vehicle), PC (Positive control; hair removal + Pansidil 400 mg/kg b.w.), SH-GT 100 (hair removal + SH-GT 100 mg/kg b.w.), SH-GT 300 (hair removal + SH-GT 300 mg/kg b.w.), and SH-GT 600 (hair removal + SH-GT 600 mg/kg b.w.). Test materials were orally administered once daily for 28 days. All values are presented as mean ± SD (*n* = 9). Different letters indicate significant differences among groups as determined by one-way ANOVA followed by Duncan’s multiple range test (*p* < 0.05).

To determine whether SH-GT modulates inhibitory signaling associated with hair follicle regression, the expression of BMP/Smad pathway-related proteins was analyzed in dorsal skin tissues (Figure 5A). The phosphorylation level of Smad2 (phospho-Smad2/Smad2) was significantly reduced in SH-GT-treated groups compared to the control group. Similarly, the expression of BMP4 was significantly decreased following SH-GT administration. In addition, the phosphorylation of Smad1/5, which mediates BMP signaling, was also markedly suppressed in SH-GT-treated groups in a dose-dependent manner (*p* < 0.05; Figure 5B).

These results indicate that SH-GT facilitates hair regeneration not only by activating proliferation-related signaling pathways but also by suppressing inhibitory signals mediated by the BMP/Smad axis, thereby promoting a favorable dorsal skin microenvironment for hair growth.

### 2.6. SH-GT Enhances Hair Growth-Related Factor mRNA Expression in a Hair-Removed Mouse Model

**Figure 6:**
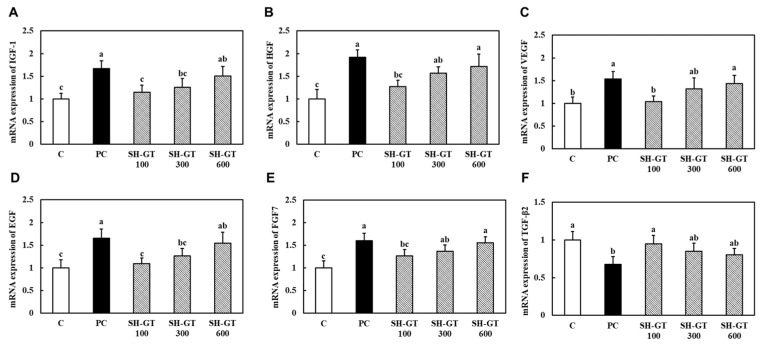
Effect of SH-GT on mRNA expression of hair growth-related factors in dorsal skin of hair-removed C57BL/6J mice. (**A**) IGF-1, (**B**) HGF, (**C**) VEGF, (**D**) EGF, (**E**) FGF7, and (**F**) TGF-β2 mRNA expression levels in dorsal skin tissues. C (Control; hair removal + vehicle), PC (Positive control; hair removal + Pansidil 400 mg/kg b.w.), SH-GT 100 (hair removal + SH-GT 100 mg/kg b.w.), SH-GT 300 (hair removal + SH-GT 300 mg/kg b.w.), and SH-GT 600 (hair removal + SH-GT 600 mg/kg b.w.). Test materials were orally administered once daily for 28 days. All values are presented as mean ± SD (*n* = 9). Different letters indicate significant differences among groups as determined by one-way ANOVA followed by Duncan’s multiple range test (*p* < 0.05).

Finally, we investigated the mRNA expression of key growth factors involved in hair follicle development and the growth cycle in dorsal skin tissues. The expression levels of IGF-1, HGF, VEGF, EGF, and FGF7 were significantly increased in SH-GT-treated groups compared to the control group, with higher doses showing more pronounced effects (*p* < 0.05; Figure 6A–E). However, no statistically significant difference in TGF-β2 expression was observed between SH-GT-treated groups and the control group (Figure 6F). These findings suggest that SH-GT may contribute to hair regeneration by enhancing the mRNA expression of multiple hair growth-related factors in dorsal skin tissues, thereby creating a favorable microenvironment for hair growth.

## 3. Discussion

Hair regrowth is primarily driven by the transition of hair follicles into the anagen phase, which is characterized by active proliferation and differentiation of follicular cells [20]. In the present study, SH-GT-treated mice exhibited accelerated hair regrowth, as evidenced by increased skin pigmentation and expanded hair growth area. In C57BL/6J mice, dorsal skin pigmentation is closely associated with hair cycle progression because follicular melanogenesis is activated during the anagen phase. In non-albino mice, telogen skin appears relatively pink, whereas anagen skin becomes darker due to melanin production in hair follicles [21]. Therefore, the increased skin pigmentation observed in SH-GT-treated mice likely reflects depilation-induced anagen entry and activation of follicular melanogenesis, which is consistent with the observed increase in hair growth area.

These phenotypic changes are well-recognized indicators of anagen induction, indicating that SH-GT facilitates the activation of hair follicle cycling. Histological analysis further supported these observations, showing that SH-GT increased dermal thickness and the number of visible hair follicle structures. During anagen progression, hair follicles elongate and extend deeper into the dermis and subcutaneous region, accompanied by active epithelial proliferation and remodeling of the surrounding dermal microenvironment. Therefore, increased dermal thickness and a higher number of visible follicular structures may reflect anagen-associated follicular activation and dermal remodeling. However, these histological findings should not be interpreted as direct evidence of newly formed hair follicles because the counting method did not distinguish de novo follicle formation from pre-existing follicles that re-entered the anagen phase after depilation. At the molecular level, SH-GT markedly enhanced the expression of proliferation-related proteins, including PCNA and Cyclin D1, in dorsal skin tissues, suggesting increased cell cycle-related activity associated with hair regrowth [22,23]. In particular, SH-GT activated Wnt/β-catenin signaling, as demonstrated by the upregulation of Wnt10b and increased levels of active β-catenin. The Wnt/β-catenin pathway is a key regulator of anagen entry and hair follicle development, and its activation has been consistently associated with enhanced hair regeneration [5,6]. In addition to Wnt signaling, SH-GT significantly enhanced the PI3K/Akt/mTOR pathway, which regulates cell survival, proliferation, and protein synthesis [9,24]. These changes suggest that SH-GT may support proliferation- and growth-related signaling in the dorsal skin microenvironment during hair regrowth. Together, activation of Wnt/β-catenin signaling may support anagen entry and epithelial proliferation, whereas PI3K/Akt/mTOR activation may further sustain cell survival, growth, and protein synthesis during active hair regrowth. Thus, these pathways may cooperatively contribute to an anagen-supportive dorsal skin microenvironment.

Importantly, SH-GT also suppressed inhibitory signaling pathways associated with hair follicle regression. The BMP/Smad axis is known to induce catagen transition and inhibit hair follicle activation [10]. In the present study, SH-GT significantly reduced the expression of BMP4 and decreased the phosphorylation of Smad1/5 and Smad2, suggesting attenuation of both BMP- and TGF-β-mediated inhibitory signaling. This dual suppression of inhibitory pathways, together with activation of proliferative signaling, indicates that SH-GT promotes hair regeneration through a balanced regulatory mechanism. Furthermore, SH-GT increased the expression of multiple growth factors, including IGF-1, HGF, VEGF, EGF, and FGF7, which are known to support hair follicle proliferation, angiogenesis, and epithelial cell activity [25,26,27]. These factors may contribute to the maintenance of the anagen phase by enhancing the local microenvironment required for hair growth.

Collagen peptides have been widely studied for their beneficial effects on skin health, including improvements in dermal structure, elasticity, and hydration [11,12]. Despite these potential benefits, studies investigating the role of orally administered collagen peptides in hair growth remain limited. The present study provides novel evidence that low-molecular-weight fish collagen peptides can promote hair regeneration by simultaneously activating proliferation-related pathways and suppressing inhibitory signaling. These findings extend the functional scope of collagen beyond skin health and suggest its potential application as a functional ingredient for improving hair growth.

In this study, Pansidil was used as the positive control because the experimental design was based on an oral hair regrowth model to evaluate the hair growth-promoting potential of SH-GT as a functional ingredient. The present study was not designed as an androgenetic alopecia model or an anti-androgen/DHT-targeted therapeutic study, but rather to determine whether orally administered SH-GT could improve hair regrowth-associated phenotypes and the dorsal skin microenvironment after hair removal. Although minoxidil is a clinically established hair growth-promoting drug, it is commonly used as a topical pharmacological treatment. Therefore, its route of administration and therapeutic context differ from those of orally administered collagen peptides. For this reason, Pansidil was selected as an oral positive control suitable for comparison with orally administered SH-GT. The dose of Pansidil used in the present study (400 mg/kg body weight) was selected based on a previous rodent hair-health study in which Pansidil was orally administered at the same dose as a positive control in an anagen-synchronized mouse model [28]. Pansidil is a multi-component oral product containing hair-related nutritional and structural components, including medicinal yeast, keratin, L-cystine, calcium pantothenate, thiamine nitrate, and p-aminobenzoic acid [29].

However, several limitations should be considered when interpreting these findings. First, the protein and mRNA expression analyses were performed using whole dorsal skin tissues; therefore, the observed changes in signaling pathways and growth factor expression reflect the overall dorsal skin microenvironment and cannot be attributed to a specific cell population. Second, the histological analysis quantified visible hair follicle structures in H&E-stained sections and did not distinguish newly regenerated follicles from preexisting follicles that reentered the anagen phase after depilation. Third, although Pansidil was used as an oral positive control based on a previous rodent hair-health study using the same oral dose [28], Pansidil is a multi-component oral product rather than a single pharmacological reference drug with a fully defined pharmacokinetic profile in mice, and a clinically established topical drug control, such as minoxidil, was not included. Fourth, only male mice were used, and the findings may not fully reflect sex-specific responses because hair follicle cycling can be influenced by sex-dependent hormonal status. Finally, complete amino acid composition and comprehensive peptide profiling of SH-GT were not performed in this study. Further studies including female mice, cell-specific validation, additional peptide characterization, and human clinical trials are needed to evaluate the broader translational relevance and peptide-specific mechanisms of SH-GT.

In conclusion, SH-GT promotes hair regrowth by enhancing proliferation-related signaling, activating Wnt/β-catenin and PI3K/Akt/mTOR pathways, suppressing BMP/Smad-mediated inhibitory signaling, and increasing the expression of hair growth-related factors in dorsal skin tissues. These findings suggest that low-molecular-weight fish collagen peptides may serve as a promising functional material for improving hair growth and supporting a dorsal skin microenvironment favorable for hair regrowth.

## 4. Materials and Methods

### 4.1. Materials and Reagents

SH-GT was provided by GELTECH (Busan, Republic of Korea), and its preparation and characterization are described in Section 4.2. Pansidil, used as the positive control, was obtained from DongKook Pharmaceutical Co., Ltd. (Seoul, Republic of Korea). Carboxymethyl cellulose (CMC) was used as the vehicle for oral administration. A depilatory cream was used for dorsal hair removal after clipping. For histological analysis, dorsal skin tissues were fixed in 10% neutral-buffered formalin and stained with hematoxylin and eosin (H&E). For Western blot analysis, lysis buffer containing a protease inhibitor, Protein Assay Dye Reagent Concentrate (Bio-Rad, Hercules, CA, USA), polyvinylidene fluoride (PVDF) membranes, and enhanced chemiluminescence (ECL; Amersham Pharmacia Biotech, Buckinghamshire, UK) were used. For real-time PCR analysis, total RNA was extracted using an RNeasy Mini Kit (QIAGEN, Gaithersburg, MD, USA), and cDNA was synthesized using the iScript cDNA Synthesis Kit (Bio-Rad). Quantitative real-time PCR was performed using iQ SYBR Green Supermix (Bio-Rad). Detailed information on the antibodies used for Western blot analysis is provided in Table S2.

### 4.2. Preparation and Characterization of SH-GT

The collagen used in this study was purified fish scale collagen derived from tilapia (*Oreochromis* genus) gelatin, which was obtained from GELTECH (Busan, Republic of Korea). The low-molecular-weight fish collagen peptide (SH-GT) was prepared using the same procedure as our previous study [13]. In brief, tilapia scale-derived collagen was obtained through acid treatment and hot-water extraction, followed by purification processes including filtration and ion exchange. The purified collagen extract was subsequently concentrated, sterilized, dried, and milled to produce tilapia gelatin. To generate low-molecular-weight collagen peptides, the gelatin was solubilized and subjected to protease-mediated enzymatic hydrolysis. The hydrolyzed peptide solution was then deodorized using activated carbon treatment and further purified through sequential filtration. After high-temperature sterilization at 120 ± 5 °C for 10–20 s, the peptide preparation was spray-dried at 170–230 °C under 150–230 bar and sieved to obtain SH-GT powder with an average particle size of 50–150 μm.

Based on the physicochemical characterization reported in our previous study [13], SH-GT contains 0.93 mg/g of the octapeptide Val-Gly-Pro-Hyp-Gly-Pro-Ala-Gly, corresponding to a molecular weight of 667.7 Da. This sequence includes collagen-characteristic amino acid residues, such as glycine, proline, and hydroxyproline. These features support the characterization of SH-GT as a tilapia fish scale-derived low-molecular-weight collagen peptide preparation.

### 4.3. Animals and Experimental Design

Male C57BL/6J mice (4-week-old males; Saeronbio Inc., Uiwang, Republic of Korea) were used for the hair regrowth study. Male mice were selected to maintain consistency with the hair-removed mouse model and to reduce potential variability associated with sex-dependent hormonal fluctuations. Although androgen-related mechanisms were not the primary focus of the present study, the use of male mice provided a consistent experimental background for evaluating the hair growth-promoting effects of orally administered SH-GT. Animals were housed under controlled environmental conditions at 23 ± 2 °C, with a 12 h light/dark cycle and relative humidity of 50 ± 5%, and were allowed free access to food and water throughout the experimental period. After a 1-week acclimatization period, the mice were randomly assigned to five groups (*n* = 9 per group): C (control; hair removal + vehicle), PC (positive control; hair removal + Pansidil, 400 mg/kg body weight), SH-GT 100 (hair removal + SH-GT, 100 mg/kg body weight), SH-GT 300 (hair removal + SH-GT, 300 mg/kg body weight), and SH-GT 600 (hair removal + SH-GT, 600 mg/kg body weight). SH-GT and Pansidil were orally administered once daily for 28 days. The dose of Pansidil (400 mg/kg body weight) was selected based on a previous rodent hair-health study in which Pansidil was orally administered at the same dose as a positive control in an anagen-synchronized mouse model [28].

On experimental day 7, the hair on the dorsal skin of all mice was removed using an animal clipper, followed by the application of a depilatory cream to the same area to remove residual hair shafts. This depilation procedure was performed to synchronize hair-cycle activation. Hair regrowth was monitored weekly after depilation. Three weeks after hair removal, corresponding to experimental day 28, all animals were sacrificed, and serum and dorsal skin tissues were collected for subsequent analyses.

During the experimental period, the general health status of the mice was monitored by measuring body weight, cage-level food consumption, and food efficiency ratio (FER), as well as by visually observing general appearance, grooming behavior, spontaneous activity, posture, and abnormal clinical signs. Food consumption was measured at the cage level and expressed as the average daily intake per mouse. FER was calculated as follows: FER = {body weight gain during the experimental period (g)/cumulative food consumption during the same period (g)} × 100. At the end of the experiment, the weights of major organs, including liver, kidney, and spleen, were also measured. No abnormal clinical signs were observed during the experimental period. A schematic overview of the experimental design, including acclimatization, oral administration, hair removal, weekly monitoring, sacrifice, and sample collection, is provided in Figure S1.

### 4.4. Evaluation of Hair Regrowth

Hair regrowth was visually monitored and photographed on experimental days 7, 14, 21, and 28, corresponding to 0, 7, 14, and 21 days after hair removal, respectively. Skin color and the extent of hair regrowth were assessed on experimental day 28, corresponding to 21 days after hair removal. Skin color score was evaluated based on the degree of dorsal skin pigmentation, which reflects the transition of hair follicles into the anagen phase. Hair growth area was quantified using ImageJ software (version 1.54d; National Institutes of Health, Bethesda, MD, USA) and calculated as follows:

Hair growth area (%) = (hair-regrown area/total depilated dorsal skin area) × 100.

The total depilated dorsal skin area was defined as the region from which hair was initially removed, and the hair-regrown area was defined as the visibly hair-covered region within the same dorsal area.

### 4.5. Histological Analysis

At the end of the experimental period, dorsal skin tissues were collected, fixed in 10% neutral-buffered formalin, embedded in paraffin, sectioned longitudinally, and stained with hematoxylin and eosin (H&E). Histological images were obtained using a light microscope. Dermal thickness was measured from the epidermal–dermal junction to the dermal–subcutaneous boundary, and the number of hair follicles was counted in each section under the same magnification. The number of hair follicles was quantified by counting visible hair follicle structures in H&E-stained longitudinal dorsal skin sections within the same defined microscopic field or region of interest for each sample. This count represents the number of histologically visible hair follicle structures in the analyzed section and does not distinguish newly regenerated follicles from pre-existing follicles that re-entered the anagen phase after depilation.

### 4.6. Western Blot Analysis

Western blot analysis was performed to evaluate the effects of SH-GT on the expression of proteins involved in cell proliferation, hair growth-promoting signaling, and hair growth-inhibitory signaling in dorsal skin tissues. At the end of the experimental period (experimental day 28, corresponding to 21 days after hair removal), dorsal skin tissues were collected from the depilated region and stored at −80 °C until protein extraction. Total protein was extracted from dorsal skin tissues using lysis buffer containing a protease inhibitor. The tissues were homogenized and incubated on ice, followed by centrifugation at 14,000 rpm for 20 min at 4 °C. The supernatants were collected for protein analysis. Protein concentrations were determined with the Bradford method using bovine serum albumin (BSA) as the standard and Protein Assay Dye Reagent Concentrate (Bio-Rad, Hercules, CA, USA). Equal amounts of protein (30 μg per sample) were separated by 10% sodium dodecyl sulfate-polyacrylamide gel electrophoresis (SDS-PAGE) and transferred onto polyvinylidene fluoride (PVDF) membranes. The membranes were blocked with 5% skim milk in TBS-T buffer (TBS containing 0.5% Tween 20) for 1 h and then incubated with primary antibodies at 4 °C for 18 h. The primary antibodies used in this study included antibodies against PCNA, Cyclin D1, Wnt10b, non-phosphorylated β-catenin, β-catenin, *p*-PI3K, PI3K, *p*-Akt, Akt, *p*-mTOR, mTOR, *p*-Smad2, Smad2, BMP4, *p*-Smad1/5, Smad1, and β-actin. Phosphorylated and active-form antibodies were diluted 1:800, other primary antibodies were diluted 1:1000, and β-actin was diluted 1:3000.

After washing three times with TBS-T buffer for 10 min each, the membranes were incubated with horseradish peroxidase-conjugated secondary antibodies (Cell Signaling Technology, Danvers, MA, USA) for 1 h. Immunoreactive bands were visualized using enhanced chemiluminescence (ECL; Amersham Pharmacia Biotech, Buckinghamshire, UK) and captured using an Ez-Capture II system (ATTO, Tokyo, Japan). Band intensities were quantified using ImageJ software (National Institutes of Health, Bethesda, MD, USA). Phosphorylated proteins were expressed relative to their corresponding total protein levels, and other proteins were normalized to β-actin.

### 4.7. Total RNA Extraction and Real-Time PCR

Total RNA was extracted from dorsal skin tissues using a commercial RNA extraction kit (QIAGEN, Gaithersburg, MD, USA) according to the manufacturer’s instructions. RNA concentration and purity were determined using a NanoDrop spectrophotometer (Thermo Fisher Scientific, Waltham, MA, USA). Complementary DNA was synthesized from purified total RNA using the iScript cDNA Synthesis Kit (Bio-Rad, Hercules, CA, USA). Quantitative real-time PCR was performed using a CFX Connect Real-Time PCR Detection System (Bio-Rad) with iQ SYBR Green Supermix and gene-specific primers. Amplification involved 40 cycles of 95 °C for 30 s, 56 °C for 30 s, and 72 °C for 45 s. Relative gene expression was calculated after normalization to the internal control gene. The primer sequences used in this study are listed in Table 1.

### 4.8. Statistical Analysis

All data are presented as mean ± standard deviation (SD). Statistical analyses were performed using one-way analysis of variance (ANOVA) followed by Duncan’s multiple range test for post hoc comparisons. Differences were considered statistically significant at *p* < 0.05. Statistical analyses were conducted using SPSS PASW Statistics software (version 23.0; SPSS Inc., Chicago, IL, USA).

## Figures and Tables

**Table 1:** Primer sets used for real-time RT-PCR.

Gene	Forward Sequence (5′-3′)	Reverse Sequence (5′-3′)
IGF-1 F (NM_010512.5)	TTC AAC AAG CCC ACA GGC TAT	ACA CTC ATC CAC AAT GCC TGT CT
HGF (BC119228.1)	AGC TTG GCT TGG CAT CCA	TTT AAG ATC TGC TTG CGC TTC TC
VEGF (NM_001287056.1)	CCA GAC CTC TCA CCG GAA AG	CTG TCA ACG GTG ACG ATG ATG
EGF (BC060741.1)	GGA CTC GGA AGC AGC TAT CAA	TCC GCT TGG CTC ATC ACA
FGF7 (NM_008008.4)	AAG GGA CCC AGG AGA TGA AGA	CAA CTG CCA CGG TCC TGA T
TGF-β2 (NM_009367.4)	AGA GCT CGA GGC GAG ATT TG	TTC TGA TCA CCA CTG GCA TAT GT
GAPDH (NM_008084)	CAT GGC CAT ATC CGT GTT CCT A	GCG GCA CGT CAG ATC CA

## Data Availability

The original contributions presented in this study are included in the article/Supplementary Material. Further inquiries can be directed to the corresponding author.

## Supplementary Materials

The following supporting information can be downloaded at: https://www.mdpi.com/article/10.3390/md24070233/s1, Table S1: Body weight, cage-level food consumption, food efficiency ratio, and major organ weights in mice treated with SH-GT; Figure S1: Schematic overview of the animal experimental design; Table S2: Antibodies used for Western blot analysis.
