# Supplementary material for: Low-Molecular-Weight Fish Collagen Peptide Enhances Hair Regrowth via Activation of Proliferative Signaling and Suppression of Inhibitory Pathways
Source: Mar Drugs. 2026 Jul 3;24(7):233. doi: 10.3390/md24070233 (PMC13412196; doi:10.3390/md24070233)
Supplement: Supplementary file 1 [file marinedrugs-24-00233-s001.zip › marinedrugs-4333230-supplementary.pdf]

Table S1. Body weight, cage-level food consumption, food efficiency ratio, and major organ weights in mice treated with SH-GT.

Measurements	C	PC	SH-GT 100	SH-GT 300	SH-GT 600
Initial body weight (g)	20.11 ± 1.36[ns]	19.66 ± 0.59	19.89 ± 1.23	19.94 ± 0.93	19.62 ± 1.33
Final body weight (g)	26.03 ± 2.10[ns]	25.64 ± 1.08	26.12 ± 1.58	25.85 ± 1.13	25.36 ± 1.52
Weight gain (g) [1)]	5.92 ± 1.78[ns]	5.98 ± 1.26	6.23 ± 1.33	5.92 ± 1.38	5.74 ± 1.27
Food consumption (g/day)	2.54 ± 0.11[b]	2.62 ± 0.22[ab]	2.62 ± 0.12[ab]	2.81 ± 0.21[a]	2.83 ± 0.21[a]
FER [2)]	5.55 ± 1.67[ns]	5.44 ± 1.14	5.67 ± 1.21	5.01 ± 1.16	4.83 ± 1.07
Liver	0.86 ± 0.09[ns]	0.85 ± 0.04	0.83 ± 0.05	0.89 ± 0.05	0.85 ± 0.07
Kidney	0.27 ± 0.02[ns]	0.27 ± 0.01	0.27 ± 0.01	0.27 ± 0.01	0.27 ± 0.01
Spleen	0.06 ± 0.01[ns]	0.06 ± 0.00	0.06 ± 0.01	0.06 ± 0.01	0.06 ± 0.02

Values are presented as mean ± SD. Food consumption was measured at the cage level and expressed as the average daily intake per mouse. FER was calculated as follows: FER (%) = [body weight gain during the experimental period (g) / cumulative food consumption during the same period (g)] × 100. Different letters indicate significant differences among groups ($p < 0.05$). ns indicates no significant difference.

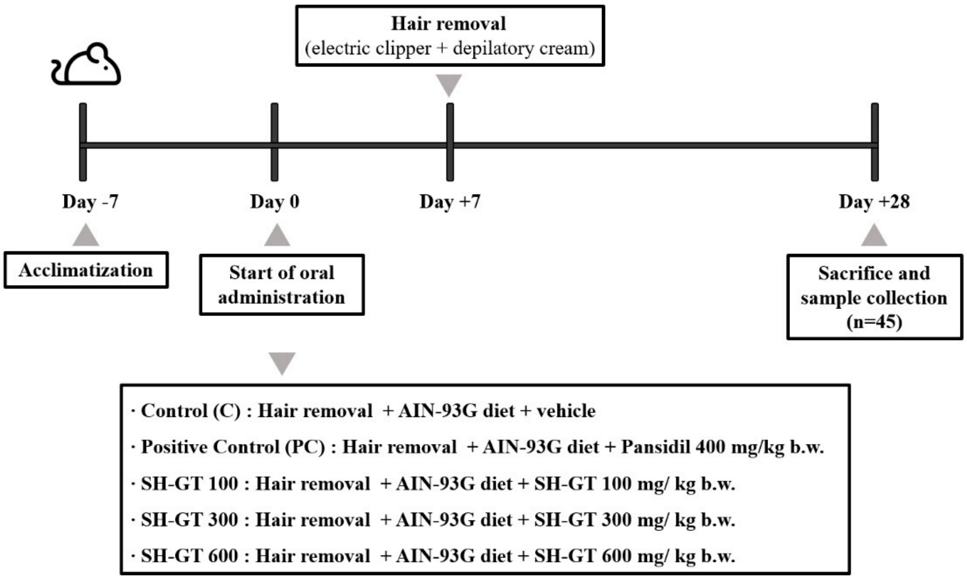

Figure S1. Schematic overview of the animal experimental design. After a 1-week acclimatization period, oral administration of Pansidil or SH-GT was started on experimental day 0 and continued once daily for 28 days. On experimental day 7, dorsal hair was removed using an electric clipper and depilatory cream. Hair regrowth was monitored weekly after depilation. On experimental day 28, corresponding to 21 days after hair removal, mice were sacrificed, and serum and dorsal skin tissues were collected for subsequent analyses. C, control; PC, positive control; b.w., body weight.

Table S2. Antibodies used for Western blot analysis.

Target protein	Antibody name	Manufacturer	Catalog No.	Dilution	Sample type
PCNA	PCNA (D3H8P) Rabbit Monoclonal Antibody	Cell Signaling Technology	13110S	1:1000	Dorsal skin
Cyclin D1	Cyclin D1 (E3P5S) Rabbit Monoclonal Antibody	Cell Signaling Technology	55506S	1:1000	Dorsal skin
Wnt10b	anti-WNT10B antibody :: Rabbit anti-Human, Mouse Wnt10B Polyclonal Antibody	Mybiosource	MBS625543	1:1000	Dorsal skin
np-β-catenin(active)	Non-phospho (Active) beta-Catenin (Ser33/37/Thr41) (D13A1) Rabbit Monoclonal Antibody	Cell Signaling Technology	8814S	1:800	Dorsal skin
β-catenin(total)	beta-Catenin (D10A8) Rabbit Monoclonal Antibody	Cell Signaling Technology	8480S	1:1000	Dorsal skin
p-PI3K	Phospho-PI3 Kinase p85 (Tyr458)/p55 (Tyr199) Antibody	Cell Signaling Technology	4228S	1:800	Dorsal skin
PI3K	PI3 Kinase p85 Antibody	Cell Signaling Technology	4292S	1:1000	Dorsal skin
p-Akt	Phospho-Akt (Ser473) Antibody	Cell Signaling Technology	9271S	1:800	Dorsal skin
Akt	Akt Antibody	Cell Signaling Technology	9272S	1:1000	Dorsal skin
p-mTOR	Phospho-mTOR (Ser2448) Antibody	Cell Signaling Technology	2971S	1:800	Dorsal skin
mTOR	mTOR Antibody	Cell Signaling Technology	2972S	1:1000	Dorsal skin
p-SMAD2	Phospho-SMAD2 (Ser465/Ser467) (E8F3R) Rabbit Monoclonal Antibody	Cell Signaling Technology	18338A	1:800	Dorsal skin
SMAD2	SMAD2 (D43B4) Rabbit Monoclonal Antibody	Cell Signaling Technology	5339A	1:1000	Dorsal skin
BMP4	Anti-BMP4 antibody	Abcam	ab39973	1:1000	Dorsal skin
p-SMAD1/5	Phospho-SMAD1/5 (Ser463/465) (41D10) Rabbit Monoclonal Antibody	Cell Signaling Technology	9516S	1:800	Dorsal skin
SMAD1	SMAD1 Antibody	Cell Signaling Technology	9743S	1:1000	Dorsal skin
β-actin	Cytoskeletal Actin Polyclonal Antibody	Bethyl Laboratories	A300-491A	1:3000	Dorsal skin
